# Immunogenicity and Safety of the M72/AS01_E_ Candidate Vaccine Against Tuberculosis: A Meta-Analysis

**DOI:** 10.3389/fimmu.2019.02089

**Published:** 2019-09-03

**Authors:** Zhenhua Ji, Miaomiao Jian, Taigui Chen, Lisha Luo, Lianbao Li, Xiting Dai, Ruolan Bai, Zhe Ding, Yunfeng Bi, Shiyuan Wen, Guozhong Zhou, Manzama-Esso Abi, Aihua Liu, Fukai Bao

**Affiliations:** ^1^Department of Microbiology and Immunology, Kunming Medical University, Kunming, China; ^2^Department of Biochemistry and Molecular Biology, Kunming Medical University, Kunming, China; ^3^Yunnan Province Key Laboratory for Tropical Infectious Diseases in Universities, Kunming Medical University, Kunming, China; ^4^The Institute for Tropical Medicine, Kunming Medical University, Kunming, China; ^5^Yunnan Province Key Laboratory for Major Childhood Diseases, Kunming Medical University, Kunming, China

**Keywords:** vaccine, tuberculosis, M72/AS01_E_, immunogenicity, safety

## Abstract

**Background:** Currently, there is no tuberculosis (TB) vaccine recommended for use in latent TB infections and healthy adults. M72/AS01_E_ is a new peptide vaccine currently under development, which may improve protection against TB disease. This vaccine has been investigated in several phase I/II clinical trials. We conducted a meta-analysis to clarify the immunogenicity and safety of the M72/AS01_E_ peptide vaccine.

**Methods:** We searched the PubMed, Embase, and Cochrane Library databases for published studies (until December 2018) investigating this candidate vaccine. A meta-analysis was performed using the standard methods and procedures established by the Cochrane Collaboration.

**Results:** Seven eligible studies—involving 4,590 participants—were selected. The analysis revealed a vaccine efficacy was 57.0%, significantly higher abundance of polyfunctional M72-specific CD4^+^ T cells [standardized mean difference (SMD) = 2.58] in the vaccine group vs. the control group, the highest seropositivity rate [relative risk (RR) = 74.87] at 1 month after the second dose of vaccination (Day 60), and sustained elevated anti-M72 IgG geometric mean concentration at study end (Day 210) (SWD = 4.94). Compared with the control, participants who received vaccination were at increased risk of local injection site redness [relative risk (RR) = 5.99], local swelling (RR = 7.57), malaise (RR = 3.01), and fatigue (RR = 3.17). However, they were not at increased risk of headache (RR = 1.57), myalgia (RR = 0.97), and pain (RR = 3.02).

**Conclusion:** The M72/AS01_E_ vaccine against TB is safe and effective. Although the vaccine is associated with a mild adverse reaction, it is promising for the prevention of TB in healthy adults.

## Introduction

Tuberculosis (TB) is the deadliest infectious disease worldwide. In 2017, there were an estimated 10 million new cases of TB and 1.6 million deaths caused by the disease (1). Treatment of latent TB infections (LTBI) and vaccination of children with the bacillus Calmette–Guérin (BCG) vaccine are the currently available effective interventions for the prevention of new infections with *Mycobacterium tuberculosis* (*M. tuberculosis*) and progression to TB disease. The only licensed BCG vaccine against TB induces protective memory that lasts for 10–20 years (1–3). However, BCG does not offer substantial protection against *M. tuberculosis* in adolescents and adults. Notably, the timeframe for the waning of BCG-induced protection throughout childhood and early adult life coincides with a gradual increase in the incidence of TB (4). In recent years, the incidence of TB has increased due to human immunodeficiency virus (HIV)/TB co-infection, and the emergence of multidrug-resistant TB and extremely drug-resistant TB. Therefore, the development of a novel safe and effective TB vaccine is urgently warranted.

The M72/AS01_E_ candidate vaccine is composed of the M72 antigen—a recombinant fusion protein derived from the two highly immunogenic *M. tuberculosis* antigens Mtb32A (Rv0125 encoding PepA) (5) and Mtb39A (Rv 1196 encoding PPE 18) (6)—and the liposome-based AS01_E_. Mtb32A and Mtb39A—present in both *M. tuberculosis* and BCG—were selected based on their ability to stimulate T-cell responses (5–9). AS01_E_ contains two immunostimulants, namely monophosphoryl lipid A and *Quillaja saponaria* Molina fraction 21, which known to mediate cytotoxic lymphocyte cells and TLR4 receptors (10). The M72/AS01_E_ has shown promising results in clinical trials involving adults and adolescents infected with *M. tuberculosis*. These studies demonstrated that the vaccine exhibited a clinically acceptable safety profile and elicited high magnitude M72-specific humoral and CD4^+^ T-cell responses (11–17). The purpose of the present analysis was to evaluate the safety and immunogenicity of two doses of the M72/AS01_E_ vaccine in HIV-positive (HIV^+^) or HIV-negative (HIV^−^) *M. tuberculosis*-infected adults and adolescents, as well as BCG-vaccinated infants.

## Methodology

### Eligibility Criteria

In this analysis, we evaluated randomized clinical trials (RCTs) of all phases investigating the M72/AS01_E_ candidate TB vaccine and including a control group (e.g., placebo, adjuvant, or other vaccines). The selection criteria for studies included the evaluation of at least one outcome related to efficacy, immunogenicity, and safety of the vaccine in different populations, and the intramuscular administration of two doses of M72/AS01_E_ or control (1 month apart). Simultaneously, our populations of interest involved HIV-infected or TB-infected individuals. Of note, we excluded studies that did not report outcomes of interest and other types of research. The first outcome of interest was the serotype-specific M72/AS01_E_ antibody response which was considered protective. The secondary outcome was the occurrence of adverse effects related to the candidate vaccine.

### Literature Search and Data Extraction

A sensitive and systematic search was performed by two independent investigators (Z.H.J. and M.M.J.). A comprehensive literature search of the PubMed, Embase, and Cochrane Library databases was conducted to identify articles published until December 2018. The key search terms were “M72/AS01_E_” or “M72/AS01” or “tuberculosis vaccine” and “immunogenicity” or “safety.” The language was restricted to English (Supplementary Material 1).

Data extraction was performed by two investigators based on the predefined inclusion and exclusion criteria. A data extraction sheet was developed based on the Cochrane Handbook for Systematic Reviews of Intervention (18) and Meta-analyses (PRISMA) guidelines (Supplementary Material 2) (19). A third investigator (Z.D.) analyzed any dissimilar results to resolve discrepancies. The data extracted included the name of the first author, date of publication, experimental design, country, study population, age, gender, follow-up, groups, immunogenicity, and safety.

### Quality of Evidence and Risk of Bias

We assessed the risk of bias for each included study using the methodology established by the Cochrane Collaboration (20). The extent to which a Cochrane review can draw conclusions regarding the effects of an intervention depends on the validity of the data obtained from the included studies. This comprises a judgement and support of the judgement for each entry in a “risk of bias table,” in which each entry addresses a specific feature of the study. The judgement for each entry determines the risk of bias as “low risk,” “high risk,” or “unclear risk.” The last category indicates either lack of information or uncertainty over the potential for bias.

### Statistical Analysis

The data were collected using Microsoft Excel (Microsoft Corp. Albuquerque, NM, USA). The Stata/SE (StataCorp, College Station, TX, USA), RevMan 5.2 (The Cochrane Collaboration, Cochrane organizations, London, UK) and GraphPad Prism 6 (GraphPad Software Inc., San Diego, CA, USA) software were used for the statistical analyses. Stata/SE was used for the meta-analysis and assessment of heterogeneity. The results were reported as relative risk (RR) and standardized mean difference (SMD) with 95% confidence intervals (95%CI). The Q and I^2^ tests were used to evaluate the statistical heterogeneity among studies. A *P* < 0.1 or *I*^2^ > 50% indicated statistically significant heterogeneity, which could be explored through regression analysis. A forest plot and funnel plot were generated to judge the overall effect size and determine the presence of publication bias. The Begg's funnel plot and Egger's test were employed for the assessment of potential publication bias. An Egger's test *P* < 0.05 was interpreted as statistically significant. Influence analysis was performed to determine the robustness of the meta-analysis. For trials including more than one treatment/control group, we used the data obtained for the combined treatment/control groups. Publication bias was evaluated using the RevMan 5.2 software and shown in the risk of bias summary diagram. Application of GraphPad Prism 6 to difference Statistics and drawing figures among groups.

### Role of the Funding Source

The funder had no role in study design, data collection, data analysis, data interpretation, or writing of the report. The corresponding author had full access to all data in the study and had final responsibility for the decision to submit for publication.

## Results

### Study Characteristics

**Figure 1:**
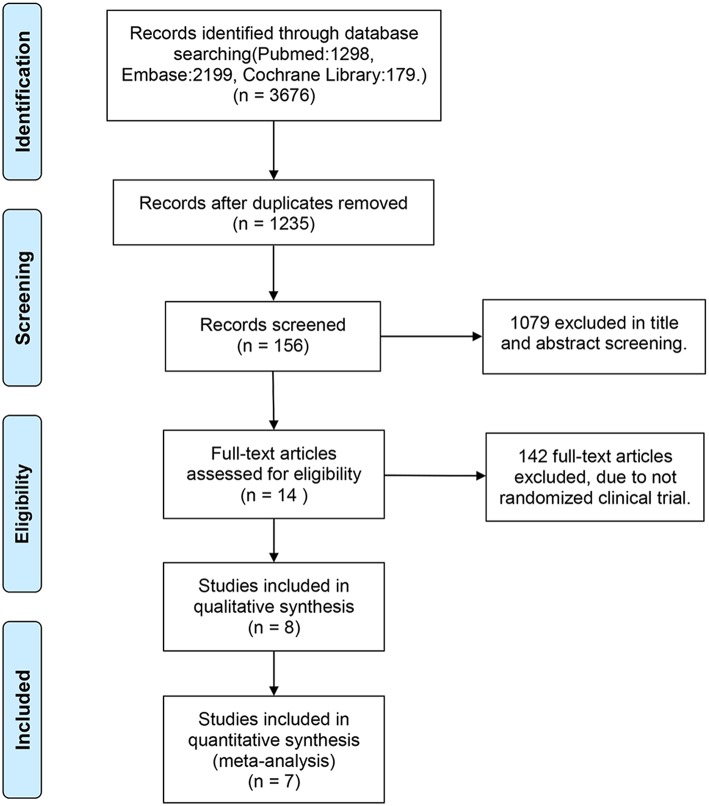
Study flow diagram.

Of the 3,676 reports identified through the systematic search, 2,441 duplicates were removed. Subsequently, we screened the titles and abstracts of the remaining studies. Of the 156 titles screened, the abstracts of 14 reports were assessed. Eventually, seven studies satisfied the standard eligibility criteria (double-blinded, two- or three-arm RCTs) (11–17). The process of study selection is illustrated in Figure 1. The main characteristics of the included studies are presented in Table 1.

**Table 1:** Characteristics of the studies included in the review.

**References**	**Design**	**Country**	**Population**	**Age**	**Male/Female**	**Followed-up**	**Groups(*N*)**
Montoya et al. (11)	Phase II RCT	Philippines	PPD-positive 3–10 mm;	18–45 years	38/142	6 months	M72/AS01B(*N* = 40) M72/AS01E(*N* = 40) M72/AS01E(*N* = 40) M72/AS02D(*N* = 40) M72/Saline(*N* = 10) AS01B alone(*N* = 10)
Thacher et al. (13)	Phase I/II RCT	Switzerland	HIV+ on cART;	18–50 years	26/11	6 months	M72/AS01E(*N* = 22) AS01 alone(*N* = 8) Saline(*N* = 7)
Idoko et al. (14)	Phase II RCT	Gambia	BCG-vaccinated infants;	2–7 months	159/141	6 months	Dose-outside EPI 1 dose M72/AS01E (*N* = 50) 2 doses M72/AS01E (*N* = 50) control(*N* = 50) Dose-within EPI 1 dose M72/AS01E (*N* = 52) 2 dosesM72/AS01E (*N* = 49) EPI only (*N* = 49)
Penn-Nicholson et al. (12)	Phase II RCT	South Africa	HIV-negative adolescents;	13–17 years	31/29	6 months	M72/AS01E(*N* = 80) Saline(*N* = 38)
Kumarasamy et al. (15)	Phase II RCT	India	QFT negative or positive;	18–59 years	167/73	12 months	ART-stable M72/AS01E (*N* = 40) Saline(*N* = 40) ART-naive M72/AS01E (*N* = 40) Saline(*N* = 40) HIV-ve M72/AS01E (*N* = 40) Saline(*N* = 40)
Gillard et al. (16)	Phase II RCT	Taiwan Estonia	Confirmed pulmonary TB; Treated pulmonary TB; No active pulmonary disease;	18–59 years	82/60	6 months	M72/AS01E (*N* = 71) Saline (*N* = 71)
Meeren et al. (17)	Phase IIb RCT	Kenya South Africa Zambia	Healthy; Stable chronic medical conditions;	18–50 years	2044/1529	3 years	M72/AS01E (*N* = 1786) Saline (*N* = 1787)

*RCT, randomized controlled trial; PPD, tuberculin purified protein derivative; BCG, Bacillus Calmette–Guerin; HIV, human immunodeficiency virus; TB, tuberculosis; cART, combination anti-retroviral therapy; QTF, QuantiFERON Gold In-Tube test*.

### Methodological Quality and Risk of Bias

Methodological quality and risk of bias were evaluated using the tool established by the Cochrane Collaboration for assessing the risk of bias. This is a two-part tool, addressing the following seven specific domains: sequence generation, allocation concealment, blinding of participants and personnel, blinding of outcome assessment, incomplete outcome data, selective outcome reporting, and “other issues.” The Cochrane Handbook provides criteria for judging the risk of bias based on the seven domains included in the tool. All included trials presented a low risk of bias for important aspects, such as random sequence generation, allocation concealment, and selective reporting. All studies followed this protocol. Unfortunately, one study did not report complete data, which resulted in a high risk of bias (Supplementary Material 3).

### Evaluation of Immunogenicity

#### Polyfunctional M72-Specific CD4^+^ T-Cell

**Figure 2:**
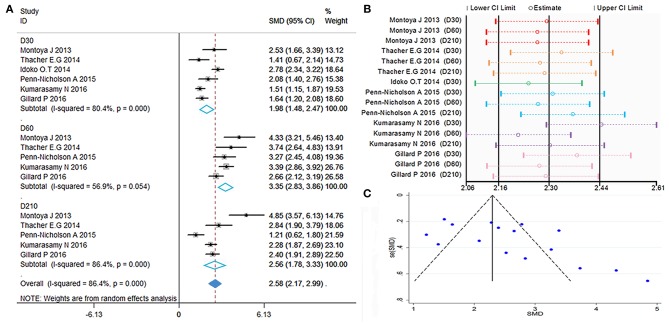
Polyfunctional M72-specific CD4^+^ T-cell evaluation. **(A)** Forest plot: a SMD > 0 indicates that vaccine can effectively stimulate the growth of polyfunctional CD4^+^ T-cells. SMD = 0, invalid result. Point estimates and 95%CI are shown for each study and for the pooled results. **(B)** Influence analysis, examine the impact of a study on the total combined effect. The vertical solid line in the middle indicates the total combined effect, while the left and right vertical solid lines indicate 95%CI. **(C)** Funnel plot: the publication bias was independently assessed by determining the symmetrical distribution of the studies.

Polyfunctional M72-specific CD4^+^ T-cells were defined as cells expressing more than two immune markers among cytokines IFN-γ, IL-2, TNF-α, CD40L, IL-17, and IL-13. The meta-analysis was performed by comparing the polyfunctional CD4^+^ T-cells of the vaccine group with those of the control group. The overall mean value of CD4^+^ T-cells was transformed using the natural logarithm (ln) form at different times (Figure 2A). Significant heterogeneity was reported (*I*^2^ > 50% and *P* < 0.1); thus, a random effects model was used.

The results showed a significant difference between the vaccinated and non-vaccinated groups in the number of polyfunctional CD4^+^ T cells at 30 days (D30) after the first dose, 60 days (D60) after the first dose (i.e., 30 days after the second dose), and 210 days (D210) after the first dose. The abundance of polyfunctional CD4^+^ T cells was higher than that reported pre-vaccination at D30 (SMD = 1.98, 95%CI = 1.48–2.47, *I*^2^ = 80.4%, *P* = 0.000), with significant boosting D60 (SMD = 3.35, 95%CI = 2.83–3.86, *I*^2^ = 56.9%, *P* = 0.054). Furthermore, the vaccinated group maintained higher levels of cytokine expression at D210 (SMD = 2.56, 95%CI = 1.78–3.33, *I*^2^ = 86.4%, *P* = 0.000). With the exception of D60, the remaining time points presented high heterogeneity.

The forest plot and funnel plot showed that the studies were symmetrically distributed (Figures 2A,C). In addition, the publication bias test showed that there was no statistically significant publication bias (Table 2). However, based on the results of the influence analysis, several articles affecting the stability of the results were identified. After removing these studies, the combined effect point estimates fell outside the total combined effect value (Figure 2B). Moreover, the regression analysis explained the main heterogeneity observed at D60 due to differences among studies. In addition, the meta-regression analysis revealed a relationship between different ages and heterogeneity at D30. The I^2^ was reduced to 20.41%, while the covariate was age (Table 3).

**Table 2:** Publication bias test.

**Test**		**CD4+ T-cell evaluation**			**CD8+ T-cell evaluation**			**IgG seropositivity rate**			**IgG GMC**			**Safety**		
		**D30**	**D60**	**D210**	**D30**	**D60**	**D210**	**D30**	**D60**	**D210**	**D30**	**D60**	**D210**	**Headache**	**Myalgia**	**Pain**
Begg's test	Z	0.75	1.22	1.22	0.00	1.22	1.22	0.00	2.63	0.34	1.70	0.00	1.04	0.34	0.00	0.00
	p	0.45	0.22	0.22	1.00	0.22	0.22	1.00	0.01^*^	0.73	0.09	1.00	0.30	0.73	1.00	1.00
Egger's test	t	0.41	1.79	1.16	−0.43	0.94	1.00	0.65	2.11	−0.17	2.30	1.43	3.76	−1.11	−0.11	−0.05
	p	0.70	0.17	0.33	0.69	0.42	0.39	0.55	0.10	0.88	0.15	0.39	0.17	0.38	0.93	0.97

GMC, IgG geometric mean concentration;

* *P < 0.05, publication bias effects were statistically significant*.

**Table 3:** Regression analysis.

**Study**	**Time**	**Covariate**	**N**	**tau2**	**I-squared res**	**Adj R-squared**
CD4^+^ T-cell evaluation	D30	Age^*^	6	0.00	20.41%	100.00%
		HIV infection	6	0.16	71.01%	36.69%
		Publication year	6	0.14	62.22%	44.50%
	D60	Adjuvant control	5	0.08	33.08%	58.90%
		Publication year	5	0.07	22.04%	63.78%
	D210	Adjuvant control	5	0.62	82.24%	53.67%
		Publication year	5	0.92	88.32%	31.71%
CD8^+^ T-cell evaluation	D30	Age	6	0.17	71.48%	51.41%
	D60	Adjuvant control	5	0.00	0.00%	91.02%
GMC	D30	Age	4	1.60	91.89%	81.35%
		Publication year	4	1.60	91.89%	81.35%
	D60	Age	3	0.19	64.33%	93.50%
		Publication year	3	0.19	64.33%	93.50%
	D210	Age	3	0.84	90.25%	44.50%
		HIV infection	3	0.56	66.72%	62.98%
		Publication year	3	0.83	90.25%	44.49%
		Sample size	3	0.00	00.00%	100%
Safety	Headache	Age	4	0.00	0.00%	100.00%
	Myalgia	HIV infection	3	0.00	0.00%	100.00%
	Pain	HIV infection	3	29.55	99.05%	11.45%
		Sample size	3	26.54	98.98%	20.49%

GMC, IgG geometric mean concentration;

* *P < 0.05, covariate effects were statistically significant; Tau2, REML estimate of between-study variance; I-squared res, % residual variation due to heterogeneity; Adj R-squared, Proportion of between-study variance explained. Age, Adjuvant control, HIV infection, Publication year, Sample size*.

### Polyfunctional M72-Specific CD8^+^ T-Cell

As previously defined, polyfunctional M72-specific CD8^+^ T-cells also express at least two immune markers of IFN-γ, IL-2, TNF-α, CD40L, IL-17, or IL-13. The initial data of polyfunctional CD8^+^ T-cells were transformed using the natural logarithm (ln), then SMD calculated, the polyfunctional CD8^+^ T-cells of the vaccine group with those of the control group were compared. The random effects model was applied, because significant heterogeneity was reported (*I*^2^ > 50% and *P* < 0.1).

**Figure 3:**
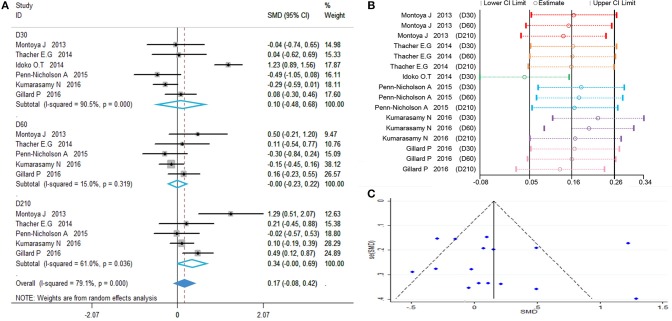
Polyfunctional M72-specific CD8^+^ T-cell evaluation. **(A)** Forest plot: a SMD > 0 indicates that vaccine can effectively stimulate the growth of polyfunctional CD8^+^ T-cells. SMD = 0, invalid result. Point estimates and 95%CI are shown for each study and for the pooled results. **(B)** Influence analysis, examine the impact of a study on the total combined effect. The vertical solid line in the middle indicates the total combined effect, while the left and right vertical solid lines indicate 95%CI. **(C)** Funnel plot: the publication bias was independently assessed by determining the symmetrical distribution of the studies.

The results are showed in Figure 3A, SMDs all contains zero at every time point, which means the number of polyfunctional CD8^+^ T-cells has no change between the vaccinated and non-vaccinated groups. Our influence analysis to the study by Idoko et al. pointed out a great influence on the total combined effect (Figure 3B), publication bias analysis showed that several studies are biased from symmetrical in the funnel plot (Figure 3C). Therefore, we did the Begg's test and Egger's test analysis, but results did not show the publication bias (Table 2). In order to find out the source of heterogeneity, we did regression analysis, the results showed that age (D30) could reduce I^2^ value in all kinds of covariables (Table 3).

### Anti-M72 IgG Seropositivity Rate

**Figure 4:**
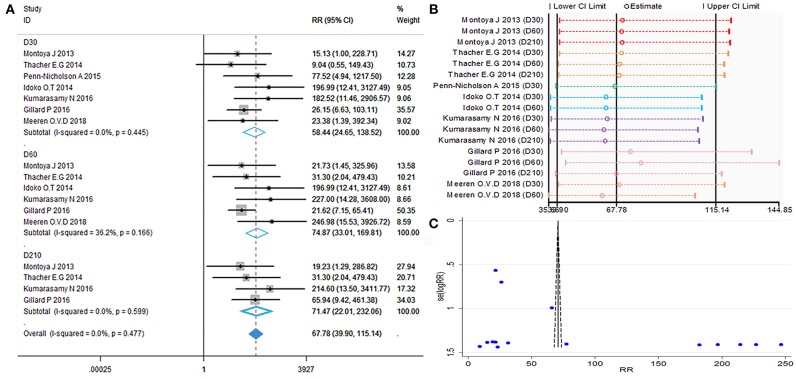
Anti-M72 IgG seropositivity rate. **(A)** Forest plot: a RR >1 indicates that the vaccine was protective. RR = 1, invalid result. Point estimates and 95%CI are shown for each study and for the pooled results. **(B)** Influence analysis, examine the impact of a study on the total combined effect. The vertical solid line in the middle indicates the total combined effect, while the left and right vertical solid lines indicate 95%CI. **(C)** Funnel plot: the publication bias was independently assessed by determining the symmetrical distribution of the studies.

Anti-M72 IgG seropositive subjects were defined as titers ≥2.8 enzyme-linked heterogeneity among the included studies (*I*^2^ < 50%, *P* > 0.1). Therefore, the fixed effect model was applied for the meta-analysis. The best results for the serotype-specific immune response were obtained 30 days after the second dose of M72/AS01_E_ (D60) (RR = 74.87, 95%CI = 33.04–169.81, *I*^2^ = 36.2%, *P* = 0.166), while the seropositivity rate RR at D60 was 74.87% (Figure 4A). These findings revealed that the probability of publication bias was exist (Table 2, Figures 4B,C). In addition, the funnel plot showed that the studies were not generally symmetrically distributed (Figure 4C).

### Anti-M72 IgG Geometric Mean Concentration (GMC)

**Figure 5:**
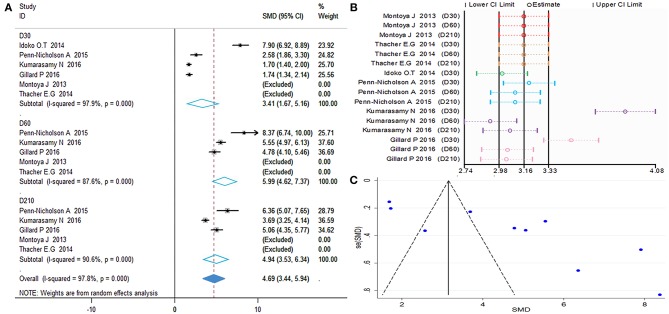
Anti-M72 IgG geometric mean concentration(GMC). **(A)** Forest plot: SMD >0 indicates that the vaccine was protective. SMD = 0, invalid result. Point estimates and 95%CI are shown for each study and for the pooled results. **(B)** Influence analysis, examine the impact of a study on the total combined effect. The vertical solid line in the middle indicates the total combined effect, while the left and right vertical solid lines indicate 95%CI. **(C)** Funnel plot: the publication bias was independently assessed by determining the symmetrical distribution of the studies.

Calculations of the anti-M72 IgG GMC were performed by taking the anti-ln of the mean of the concentration transformations. Because the heterogeneity test showed *P* = 0.000 with *I*^2^> 50%, the random effects model was applied (Figure 5). The results showed that all time point effect size was high contrast and that the second dose was the highest titer (D30 SMD = 3.41, 95%CI = 1.67–5.16, *I*^2^ = 97.9%, *P* = 0.000; D60 SMD = 5.99, 95%CI = 4.62–7.73, I^2^ = 87.6%, *P* = 0.000; D210 SMD = 4.94, 95%CI = 3.53–6.34, *I*^2^ = 90.6%, *P* = 0.000).

As shown in Figures 5A,C, the data presented by Kumarasamy N (D30, D60) (15) and Gillard P (D30) (16) significantly affected the stability of the results. The Begg and Egger tests did not show significant publication bias for the studies included in the summary analysis (Table 2). Although the regression analysis did not identify the main causes of high heterogeneity (Table 3), negligible publication bias is not a major factor. Finally, in this study, we included control subjects who received a non-adjuvanted vaccine, alum-adjuvanted vaccine, or placebo. Thus, the level of reactogenicity in the control groups may be affected.

### Evaluation of Safety

The local and systemic toxicity associated with the M72/AS01_E_ vaccine were evaluated in six studies (11–16). Grade 1 local and systemic toxicities were observed in most participants, whereas grade 3 toxicities were noted in some cases. Injection site reactions observed after vaccination with M72/AS01_E_ were redness (RR = 5.99, 95%CI = 1.45–24.85, *I*^2^ = 11.9%, *P* = 0.287), swelling (RR = 7.57, 95%CI = 2.19–26.81, *I*^2^ = 0.0%, *P* = 0.594). Adverse events (AEs) occurred more frequently in the vaccine group compared with control. The most frequently reported toxicity AEs were malaise (RR = 3.01, 95%CI = 1.02–8.92, *I*^2^ = 0.0%, *P* = 0.997) and fatigue (RR = 3.17, 95%CI = 1.43–7.05, *I*^2^ = 0.0%, *P* = 0.651). There were no differences detected between the M72/AS01_E_ and control arms in terms of headache (RR = 1.57, 95%CI = 0.71–3.50, *I*^2^ = 51.7%, *P* = 0.102), myalgia (RR = 0.97, 95%CI = 0.14–6.62, *I*^2^ = 61.0%, *P* = 0.077) and pain (RR = 3.02, 95%CI = 0.64–14.14, *I*^2^ = 87.4%, *P* = 0.000).

**Figure 6:**
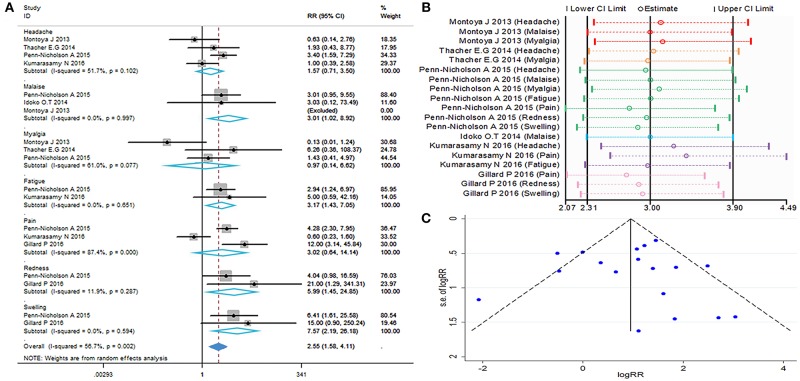
Safety evaluation. **(A)** Forest plot: a RR >1 indicates that the vaccine was protective, the result of intersection with the intermediate invalid line was invalid. RR = 1, invalid result. Point estimates and 95%CI are shown for each study and for the pooled results. **(B)** Influence analysis, examine the impact of a study on the total combined effect. The vertical solid line in the middle indicates the total combined effect, while the left and right vertical solid lines indicate 95%CI. **(C)** Funnel plot: the publication bias was independently assessed by determining the symmetrical distribution of the studies.

The meta-analysis of safety is shown in Figures 6A,B. The funnel plot (Figure 6C) and Table 2 show that evaluation of safety publication bias was excluded. Although the source of heterogeneity could not be definitively identified, it may be related to age or gender (Table 3). Individuals of different ages and genders exhibit different subjective judgments of headache and myalgia, and normal incidence is also different. This needs to be taken into consideration when interpreting the results of such evaluations.

### Subgroup Analysis

We considered that HIV-positive and HIV-negative individuals have different immunity responses to vaccines and may have an impact on the results, so we performed a subgroup analyses. Six publications included in this meta-analysis compared immune responses after vaccination in HIV-positive and HIV-negative patients, among them, two studies are related to the HIV-positive population.

**Figure 7:**
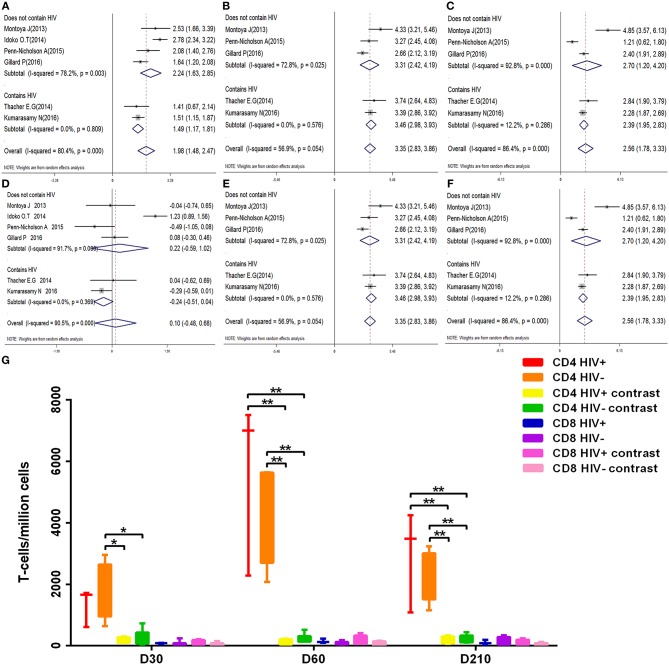
Subgroup analysis polyfunctional CD4^+^ and CD8^+^ T-cell. **(A–F)** Forest plot: a SMD > 0 indicates that vaccine can effectively stimulate the growth of polyfunctional CD4^+^ or CD8^+^ T-cells. SMD = 0, invalid result. **(A–C)** are the result of polyfunctional CD4^+^ T-cell analysis of D30, D60, and D210, respectively. **(D–F)** are the result of polyfunctional CD4^+^ T-cell analysis of D30, D60, and D210, respectively. **(G)** Box diagram: The transverse coordinates are the date of vaccination. The longitudinal coordinates are the number of polyfunctional CD4^+^ or CD8^+^ T-cells. Statistically significant differences between the two groups are indicated by star symbols: ^*^p < 0.05; ^**^p < 0.001.

We examined the kinetic changes of polyfunctional CD4^+^ T-cell and polyfunctional CD8^+^ T-cell counts both in HIV-positive and HIV-negative individuals (Figure 7G). At D30 after the first dose, compared with contrast, polyfunctional CD4^+^ T-cell counts in HIV-positive and HIV-negative populations both were higher (*p* < 0.05). The elevation in polyfunctional CD4^+^ T-cell counts was most prominent at D60 after the first dose, polyfunctional CD4^+^ T-cell counts both in HIV-positive and HIV-negative were higher than contrast (*p* < 0.01). At D210 after the first dose, polyfunctional CD4^+^ T-cell counts became relatively stable (*p* < 0.01), the count of polyfunctional CD4^+^ T-cell both in HIV-positive and HIV-negative were always higher than contrast. Importantly, there is no statistical difference in the count of polyfunctional CD4^+^ T-cell between HIV-positive and HIV-negative during vaccination (*p* > 0.05). At every time point, the number of polyfunctional CD8^+^ T-cell were no statistical difference between HIV-positive and HIV-negative individuals (*p* > 0.05). The corresponding forest map also showed no significant difference between the two groups, and the heterogeneity did not decrease after subgrouping (Figures 7A–F).

**Figure 8:**
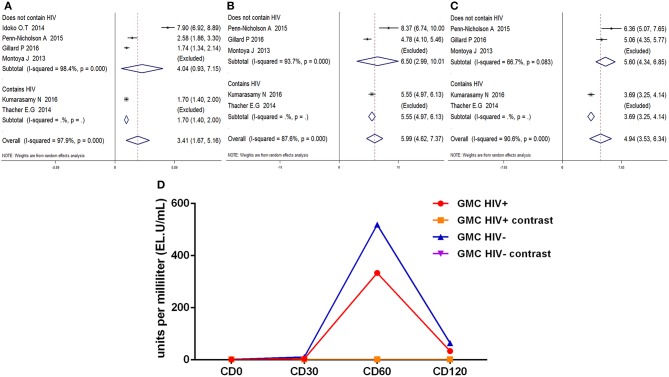
Subgroup analysis Anti-M72 antibody IgG geometric mean concentration(GMC). **(A–C)** Forest plot: SMD >0 indicates that the vaccine was protective. SMD = 0, invalid result. Point estimates and 95%CI are shown for each study and for the pooled results. **(D)** Line chart: the anti-M72 IgG GMC varies (Ordinate) with time in different time points (Abscissa) after vaccination.

Similarly, the anti-M72 IgG GMC was most prominent at D60 after the first dose, anti-M72 IgG GMC both in HIV-positive and HIV-negative groups were higher (*p* < 0.01) (Figure 8D) in comparison with contrast. At every time point, there is no statistical difference in the anti-M72 IgG GMC between HIV-positive and HIV-negative groups. In short, The results of the subgroup analyses were also similar (Figures 8A–C).

## Discussion

The elimination of TB has been partly hindered by the ability of *M. tuberculosis* to remain dormant in the human body for years without causing disease—a state referred to as LTBI (21). It is estimated that approximately one quarter of the global population are infected with *M. tuberculosis*. Of those, 5–10% will develop TB disease during their lifetime (1). Although the majority of infected individuals are asymptomatic, they develop a strong acquired immune response to the pathogen (22). Thus, the prevention and treatment of LTBI is currently the focus of ongoing research. Eradication of TB requires a highly efficacious TB vaccine. The evaluation of immunogenicity and safety of the M72/AS01E candidate vaccine may be provide rationale for further large-scale efficacy trials of M72/AS01E vaccine.

This study is the first systematic review and meta-analysis evaluating the safety and immunogenicity of candidate vaccine M72/AS01_E_ in complex individuals. In the examined data, we did not identify serious AEs related to the M72/AS01_E_ vaccine or safety concerns. The results of our meta-analysis showed that the incidence of local AEs (i.e., pain) or general AEs (i.e., headache and myalgia) were low during a 6-month follow-up study. However, redness, swelling, fatigue, and malaise were significantly higher in the vaccinated group vs. the placebo group. For the M72/AS01_E_ vaccine, solicited AEs were primarily mild to moderate in intensity and transient. These local or general AEs were limited in duration (most resolved within a few days) and there was no requirement for specific diagnostic or therapeutic interventions. The AE profiles associated with M72/AS01_E_ were clinically acceptable in antiretroviral therapy (ART)-stable and ART-naive HIV-infected individuals. Therefore, the use of M72/AS01_E_ vaccine for the prevention of TB is safe.

In vaccine clinical trials, the abundance of antigen-specific T cells and antibody concentrations are often used to explain the response to vaccination (23). Various kinds of preclinical studies have demonstrated that humoral immunity may play a part in the protection against *M. tuberculosis* (24–26). Our results showed that two-dose M72/AS01_E_ vaccination induced higher seroconversion rates vs. placebo. Meanwhile, there was a sharp increase in the seroconversion rates after the second vaccine dose compared with the first. Kumarasamy et al. reported that M72-specific antibodies induced through vaccination with M72/AS01_E_ persisted for a maximum of 3 years (27). Notably, two-dose vaccination offered more durable long-term protection. Moreover, the AS01 adjuvant system is a component of the RTS, S/AS01 malaria vaccine (28–30) and recombinant zoster vaccine (31) (both currently investigated in phase III studies). Due to its association with improvement in adaptive immune responses (humoral and cellular), AS01_E_ may promote increases in Ag-specific antibody responses, cytokine release and levels of costimulatory molecules in humans (32). Use of adjuvants is required to induce the most potent immune responses. Therefore, the use of a potent adjuvant such as AS01_E_ may allow the reduction of antigen doses (i.e., antigen sparing effect).

Based on preclinical studies, the protection against *M. tuberculosis* is mediated by antigen-specific polyfunctional CD4^+^ T cells (33–37). Although immune correlates of protection against TB have not been defined (38), IFN-γ, TNF-a, IL-2, and IL-17 are important for the control of mycobacterial infection (39–41). IFN-γ and TNF-α can activates infected macrophages, respectively, which in turn inhibit *M. tuberculosis* growth by inducing iNOS and autophagy (42, 43). Moreover, IFN-γ and TNF-α synergistically mediate killing of pathogens (42). IL-2 induces CD4^+^ and CD8^+^ T cells proliferation and differentiation, and promotes the development of memory T cells during primary infection. IL-17 plays an important antimicrobial pro-inflammatory role in the stages of *M. tuberculosis* infection by inducing neutrophil generation, stimulate cytokine production (44). Studies have shown that polyfunctional IFN-γ^+^ IL-2^+^ TNF-α^+^ CD4^+^ T cells may produce higher levels of each cytokine on a per-cell basis, compared with other T cells (37, 45).

A notable feature of M72/AS01_E_ is the complexity of the induced T-cell responses. A variety of distinct cytokine-producing populations of M72-specific CD4^+^ T-cell responses were induced after vaccination, including a discrete population of Th17 cells (46). The M72/AS01_E_ vaccine-induced CD4^+^ T-cell responses were strongly Th1-biased (7, 11, 47). Thus, the immunogenicity of TB candidate vaccines is typically expressed in terms of magnitudes of the induced specific CD4^+^ T-cell responses expressing Th1-type cytokines. Similarly, evidence showed that vaccination with M72/AS01_E_ induced robust polyfunctional M72-specific CD4^+^ T cells, which persisted for 3 years. This response was significantly boosted through a second vaccination, indicating the presence of effector memory T cells. By comparing it with other candidate vaccines, M72/AS01_E_ induced higher memory Th1 cytokine-expressing CD4 T cell responses than other novel vaccine (48).

In a small study, the abundance of polyfunctional M72-specific CD4^+^ T cells expressing CD40L^+^ TNF-α^+^ IFN-γ^+^, CD40L^+^ IL-2^+^ TNF-α^+^ IFN-γ^+^, or CD40L^+^ IFN-γ^+^ profiles was higher in the TB-treatment (adults receiving treatment for TB disease who have completed the intensive phase of treatment) and TB-treated (adults with previous history of successfully treated pulmonary TB disease groups) vs. the TB-naïve (adults who never had TB) group (16). This finding is consistent with results showing that patients with sputum smear^+^ TB exhibited decreased polyfunctional IFN-γ^+^ IL-2^+^ TNF-α^+^ and IL-2-producing specific CD4^+^ T cells and recovery of the number of T cells during therapy vs. those with sputum smear^−^ TB and LTBI (49). These data indicate that the functional capacity of *M. tuberculosis*-specific T cells elicited by the M72/AS01_E_ vaccine may also be progressively impaired with an increased mycobacterial load—subsequently recovering during therapy.

The abundance of M72-specific CD4^+^ T cells expressing IL-17 was low and did not co-express Th1 cytokines. In this analysis, almost no M72-specific IL-13^+^CD4^+^ T-cell responses were detected (data not shown). In humans, Th2-type responses (i.e., IL-4, IL-5, and IL-13) correlate with TB immunopathology and suppress the production of IFN-γ and TNF-α. Maintaining the balance between the Th1/Th2 responses may contribute to the control of *M. tuberculosis* infection. Of note, M72/AS01_E_ vaccine-induced M72-specific CD8^+^ T-cell responses were low and did not increase significantly over time in M72/AS01_E_ recipients (50).

The clinically acceptable safety, the potent and sustained T-cell responses, and the induction of a M72-specific IgG antibody after vaccination suggest that M72/AS01_E_ is a good candidate to advance into efficacy trials. A study found that—among healthy *M. tuberculosis*-infected, largely BCG-vaccinated, HIV-negative adults—M72/AS01_E_ was linked to a significantly lower incidence of pulmonary TB vs. placebo. After a mean follow-up period of 2.3 years, the efficacy of M72/AS01_E_ in preventing active pulmonary TB (based on two positive sputum tests) was 54%. Moreover, polymerase chain reaction data indicated that—in individuals with LTBI—the total vaccine efficacy was 57% (17). However, this trial focused exclusively on LTBI populations. Therefore, studies with larger sample sizes are warranted to demonstrate the efficacy of the M72/AS01_E_ vaccine in a wider population. Populations of interest may include healthy individuals without previous exposure to TB, individuals with an impaired immune system (HIV^+^ patients, diabetics, users of injectable drugs, etc.), household contacts of TB patients who with a higher risk of infection, and individuals across various geographical settings. In addition, it is important to compare the efficacy and duration of protection provided by the M72/AS01_E_ vaccine against all forms of TB in adolescents and adults vs. those associated with BCG. The TB vaccine clinical pipeline is being diversified, and new models of delivery by the pulmonary and mucosal routes examined (For example, the MVA85A is re-entering phase I trials as an aerosol vaccine), that holds promise for future development (51, 52).

Finally, the immunological analysis of study data may assist in better understanding the protective immune mechanisms against TB. The development of vaccines has been an iterative process. International collaborations are of crucial importance to offer renewed hope that effective new vaccines against TB can be developed (53, 54).

## Data Availability

All datasets generated for this study are included in the manuscript and/or the Supplementary Files.

## Author Contributions

FB, AL, ZJ, and MJ conceived and designed the study. ZJ and MJ did the database search and screening. YB, SW, GZ, and LLi did the data extraction. TC, LLu, XD, RB, and M-EA did the quality assessment. ZJ and MJ did the analysis, and in conjunction with SW and ZD interpreted the data and drafted the manuscript, in collaboration with FB and AL.

### Conflict of Interest Statement

The authors declare that the research was conducted in the absence of any commercial or financial relationships that could be construed as a potential conflict of interest.

## References

[1] World Health Organization Executive Summary. Global Tuberculosis Report 2018. Available online at: https://www.who.int/tb/publications/global_report/en/

[2] Hart PD Sutherland I. BCG and vole bacillus vaccines in the prevention of tuberculosis in adolescence and early adult life. Br Med J. (1977) 2: 293–95. 10.1136/bmj.2.6082.293 326347 PMC1630784

[3] Comstock GW Woolpert SF Livesay VT. Tuberculosis studies in Muscogee County, Georgia. Twenty-year evaluation of a community trial of BCG vaccination. Public Health Rep. (1976) 91: 276–280. 818671 PMC1439003

[4] Dockrell HM Smith SG. What have we learnt about BCG vaccination in the last 20 years? Front Immunol. (2017) 8:1134. 10.3389/fimmu.2017.01134 28955344 PMC5601272

[5] Skeiky YA Lodes MJ Guderian JA Mohamath R Bement T Alderson MR. Cloning, expression, and immunological evaluation of two putative secreted serine protease antigens of *Mycobacterium tuberculosis*. Infect Immun. (1999) 67:3998–4007. 10417166 10.1128/iai.67.8.3998-4007.1999 PMC96687

[6] Dillon DC Alderson MR Day CH Lewinsohn DM Coler R Bement T. Molecular characterization and human T-cell responses to a member of a novel *Mycobacterium tuberculosis* mtb39 gene family. Infect Immun. (1999) 67:2941–50. 10338503 10.1128/iai.67.6.2941-2950.1999 PMC96604

[7] Leroux-Roels I Forgus S De Boever F Clement F Demoitié MA Mettens P. Improved CD4(+) T cell responses to *Mycobacterium tuberculosis* in PPD-negative adults by M72/AS01 as compared to the M72/AS02 and Mtb72F/AS02 tuberculosis candidate vaccine formulations: a randomized trial. Vaccine. (2013) 31:2196–206. 10.1016/j.vaccine.2012.05.035 22643213

[8] Skeiky YAW Alderson MR Ovendale PJ Guderian JA Brandt L Dillon DC Differential immune responses and protective efficacy induced by components of a tuberculosis polyprotein vaccine, Mtb72F, delivered as naked DNA or recombinant protein. J Immunol. (2004) 172: 7618–28. 10.4049/jimmunol.172.12.7618 15187142

[9] Nathalie GO Marcelle VM Recent clinical experience with vaccines using MPL- and QS-21-containing adjuvant systems. Expert Rev Vaccines. (2011) 10:471 10.1586/erv.11.29 21506645

[10] Baldridge J Myers K Johnson D Persing D Cluff C Hershberg R Monophosphoryl Lipid A and Synthetic Lipid A Mimetics as TLR4-Based Adjuvants and Immunomodulators. Clifton: Humana Press (2006).

[11] Montoya J Solon JA Cunanan SRC Acosta L Bollaerts A Moris P. A randomized, controlled dose-finding phase II study of the M72/AS01 candidate tuberculosis vaccine in healthy PPD-positive adults. J Clin Immunol. (2013) 33:1360–75. 10.1007/s10875-013-9949-3 24142232 PMC3825318

[12] Penn-Nicholson A Geldenhuys H Burny W Most R Day CL Jongert E. Safety and immunogenicity of candidate vaccine M72/AS01E in adolescents in a TB endemic setting. Vaccine. (2015) 33: 4025–34. 10.1016/j.vaccine.2015.05.088 26072017 PMC5845829

[13] Thacher EG Cavassini M Audran R Thierry AC Bollaerts A Cohen J. Safety and immunogenicity of the M72/AS01 candidate tuberculosis vaccine in HIV-infected adults on combination antiretroviral therapy: a phase I/II, randomized trial. AIDS. (2014) 28: 1769–81. 10.1097/QAD.0000000000000343 24911353

[14] Idoko OT Owolabi OA Owiafe PK Moris P Odutola A Bollaerts A. Safety and immunogenicity of the M72/AS01 candidate tuberculosis vaccine when given as a booster to BCG in Gambian infants: an open-label randomized controlled trial. Tuberculosis. (2014) 94:564–78. 10.1016/j.tube.2014.07.001 25305000

[15] Kumarasamy N Poongulali S Bollaerts A Moris P Beulah FE Ayuk LN. A randomized, controlled safety, and immunogenicity trial of the M72/AS01 candidate tuberculosis vaccine in HIV-positive Indian adults. Medicine. (2016) 95:e2459. 10.1097/MD.0000000000002459 26817879 PMC4998253

[16] Gillard P Yang PC Danilovits M Su WJ Cheng SL Pehme L Safety and immunogenicity of the M72/AS01E candidate tuberculosis vaccine in adults with tuberculosis: a phase II randomized study. Tuberculosis. (2016) 100:118–27. 10.1016/j.tube.2016.07.005 27553419

[17] Van Der Meeren O Hatherill M Nduba V Wilkinson RJ Muyoyeta M Van Brakel E. Phase 2b controlled trial of M72/AS01E vaccine to prevent tuberculosis. N Engl J Med. (2018) 379:1621–34. 10.1056/NEJMoa1803484 30280651 PMC6151253

[18] Higgins JPT Deeks JJ Chapter 7: Selecting studies and collecting data. Cochrane Handbook for Systematic Reviews of Interventions Version 5.1.0. The Cochrane Collaboration (2011). Available online at: https://training.cochrane.org/handbook

[19] Moher D Liberati A Tetzlaff J Altman DG The PG Preferred reporting items for systematic reviews and meta-analyses: the PRISMA statement. PLoS Med. (2009) 6:e1000097 10.1371/journal.pmed.1000097 19621072 PMC2707599

[20] Higgins JPT Altman DG Sterne JAC Chapter 8: Assessing risk of bias in included studies. Cochrane Handbook for Systematic Reviews of Interventions Version 5.1.0. The Cochrane Collaboration (2011). Available online at: https://training.cochrane.org/handbook

[21] Velayati AA Abeel T Shea T Konstantinovich ZG Birren B Cassell GH. Populations of latent Mycobacterium tuberculosis lack a cell wall: isolation, visualization, and whole-genome characterization. Int J Mycobacteriol. (2016) 5:66–73. 10.1016/j.ijmyco.2015.12.001 26927992 PMC5443679

[22] Gideon HP Flynn JL. Latent tuberculosis: what the host “sees”? Immunol Res. (2011) 50: 202–12. 10.1007/s12026-011-8229-7 21717066 PMC3788603

[23] Van RDB Laurane DM Geert LR Bechtold V Clement F Coccia M Adjuvant-associated peripheral blood mRNA profiles and kinetics induced by the adjuvanted recombinant protein candidate tuberculosis vaccine M72/AS01 in bacillus Calmette-Guerin-vaccinated adults. Front immunol. (2018) 9:564 10.3389/fimmu.2018.00564 29632533 PMC5879450

[24] Glatman-Freedman A. The role of antibody-mediated immunity in defense against Mycobacterium tuberculosis: advances toward a novel vaccine strategy. Tuberculosis. (2006) 86:191–7. 10.1016/j.tube.2006.01.008 16584923

[25] Phuah JY Mattila JT Lin PL Flynn JL. Activated B cells in the granulomas of nonhuman primates infected with *Mycobacterium tuberculosis*. Am J Pathol. (2012) 181:508–14. 10.1016/j.ajpath.2012.05.009 22721647 PMC3409439

[26] Pethe K Alonso S Biet F Delogu G Brennan MJ Locht C. The heparin-binding haemagglutinin of *M. tuberculosis* is required for extrapulmonary dissemination. Nature. (2001) 412:190–4. 10.1038/35084083 11449276

[27] Kumarasamy N Poongulali S Beulah FE Akite EA Ayuk LN Bollaerts A. Long-term safety and immunogenicity of the M72/AS01E candidate tuberculosis vaccine in HIV-positive and -negative Indian adults: results from a phase II randomized controlled trial. Medicine. (2018) 97:e13120. 10.1097/MD.0000000000013120 30407329 PMC6250513

[28] Agnandji ST Lell B Soulanoudjingar SS Fernandes JF Abossolo BP Conzelmann C. First results of phase 3 trial of RTS, S/AS01 malaria vaccine in African children. N Engl J Med. (2011) 365:1863–75. 10.1056/NEJMoa1102287 22007715

[29] Agnandji ST Lell B Fernandes JF Abossolo BP Methogo BG Kabwende AL. A phase 3 trial of RTS, S/AS01 malaria vaccine in African infants. N Engl J Med. (2012) 367:2284–95. 10.1056/NEJMoa1208394 23136909 PMC10915853

[30] Moncunill G De Rosa SC Ayestaran A Nhabomba AJ Mpina M Cohen KW. RTS, S/AS01E malaria vaccine induces memory and polyfunctional T Cell responses in a pediatric African phase III trial. Front immunol. (2017) 8:1008. 10.3389/fimmu.2017.01008 28878775 PMC5572329

[31] Dendouga N Fochesato M Lockman L Mossman S Giannini SL. Cell-mediated immune responses to a varicella-zoster virus glycoprotein E vaccine using both a TLR agonist and QS21 in mice. Vaccine. (2012) 30:3126–35. 10.1016/j.vaccine.2012.01.088 22326899

[32] Livingston PO Adluri S Helling F Yao TJ Kensil CR Newman MJ. Phase 1 trial of immunological adjuvant QS-21 with a GM2 ganglioside-keyhole limpet haemocyanin conjugate vaccine in patients with malignant melanoma. Vaccine. (1994) 12: 1275–80. 10.1016/S0264-410X(94)80052-2 7856291

[33] Stenger S. Immunological control of tuberculosis: role of tumour necrosis factor and more. Ann Rheum Dis. (2005) 64:iv24–28. 10.1136/ard.2005.042531 16239381 PMC1766911

[34] Flynn JL. Immunology of tuberculosis and implications in vaccine development. Tuberculosis. (2004) 84: 93–101. 10.1016/j.tube.2003.08.010 14670350

[35] Henrik M Detjen AK Schuck SD Gutschmidt A Wahn U Magdorf K *Mycobacterium tuberculosis*-specific CD4+, IFNgamma+, and TNFalpha+ multifunctional memory T cells coexpress GM-CSF. Cytokine. (2008) 43:143–8. 10.1016/j.cyto.2008.05.002 18603443

[36] Aagaard CS Hoang TL Vingsbo-Lundberg C Dietrich J Andersen P. Quality and vaccine efficacy of CD4+ T cell responses directed to dominant and subdominant epitopes in ESAT-6 from *Mycobacterium tuberculosis*. J Immunol. (2009) 183:2659–68. 10.4049/jimmunol.0900947 19620314

[37] Lindenstrøm T Agger EM Korsholm KS Darrah PA Aagaard C Seder RA. Tuberculosis subunit vaccination provides long-term protective immunity characterized by multifunctional CD4 memory T cells. J Immunol. (2009) 182:8047. 10.4049/jimmunol.0801592 19494330

[38] Kagina BM Abel B Scriba TJ Hughes EJ Keyser A Soares A Specific T cell frequency and cytokine expression profile do not correlate with protection against tuberculosis after bacillus Calmette-Guerin vaccination of newborns. Am J Respir Crit Care Med. (2010) 182:1073–9. 10.1164/rccm.201003-0334OC 20558627 PMC2970848

[39] Keane J Gershon S Wise RP Mirabile-Levens E Kasznica J Schwieterman WD. Tuberculosis associated with infliximab, a tumor necrosis factor alpha-neutralizing agent. N Engl J Med. (2001) 345:1098–104. 10.1056/NEJMoa011110 11596589

[40] Khader SA Bell GK Pearl JE Fountain JJ Rangel-Moreno J Cilley GE. IL-23 and IL-17 in the establishment of protective pulmonary CD4+ T cell responses after vaccination and during *Mycobacterium tuberculosis* challenge. Nat Immunol. (2007) 8:369–77. 10.1038/ni1449 17351619

[41] Millington KA Innes JA Hackforth S Hinks TS Deeks JJ Dosanjh DP. Dynamic relationship between IFN-gamma and IL-2 profile of *Mycobacterium tuberculosis*-specific T cells and antigen load. J Immunol. (2007) 178:5217–26. 10.4049/jimmunol.178.8.5217 17404305 PMC2743164

[42] Lewinsohn DA Gold MC Lewinsohn DM. Views of immunology: effector T cells. Immunol Rev. (2011) 240:25–39. 10.1111/j.1600-065X.2010.00997.x 21349084

[43] Allie N Grivennikov SI Keeton R Hsu NJ Bourigault ML Court N. Prominent role for T cell-derived tumour necrosis factor for sustained control of *Mycobacterium tuberculosis* infection. Sci Rep. (2013) 3:1809. 10.1038/srep01809 23657146 PMC3648802

[44] Lyadova IV Panteleev AV T1 and T17 cells in tuberculosis: protection, pathology, and biomarkers. Mediators Inflamm. (2015) 2015:854507 10.1155/2015/854507 26640327 PMC4657112

[45] Darrah PA Patel DT De Luca PM Lindsay RW Davey DF Flynn BJ. Multifunctional TH1 cells defne a correlate of vaccine-mediated protection against *Leishmania major*. Nat Med. (2007) 13:843–50. 10.1038/nm1592 17558415

[46] Day CL Tameris M Mansoor N Rooyen M Kock M Geldenhuys H. Induction and regulation of T-cell immunity by the novel tuberculosis vaccine M72/AS01 in South African adults. Am J Respir Crit Care Med. (2013) 188:492–502. 10.1164/rccm.201208-1385OC 23306546 PMC3778736

[47] Spertini F Audran R Lurati F Ofori-Anyinam O Zysset F Vandepapelière P. The candidate tuberculosis vaccine Mtb72F/AS02 in PPD positive adults: a randomized controlled phase I/II study. Tuberculosis. (2013) 93:179–88. 10.1016/j.tube.2012.10.011 23219236

[48] Rodo MJ Rozot V Nemes E Dintwe O Hatherill M Little F. A comparison of antigen-specific T cell responses induced by six novel tuberculosis vaccine candidates. PLoS Pathog. (2019) 15:e1007643. 10.1371/journal.ppat.1007643 30830940 PMC6417742

[49] Day CL Abrahams DA Lerumo L Janse van Rensburg E Stone L O'rie T. Functional capacity of Mycobacterium tuberculosis-specific T cell responses in humans is associated with mycobacterial load. J immunol. (2011) 187:2222–32. 10.4049/jimmunol.1101122 21775682 PMC3159795

[50] Li Q Zhang H Yu L Wu C Luo X Sun H. Down-regulation of Notch signaling pathway reverses the Th1/Th2 imbalance in tuberculosis patients. Int Immunopharmacol. (2018) 54:24–32. 10.1016/j.intimp.2017.10.026 29100034

[51] Counoupas C Triccas JA Britton WJ Deciphering protective immunity against tuberculosis: implications for vaccine development. Expert Rve Vaccines. (2019) 4:1–12. 10.1080/14760584.2019.1585246 30793629

[52] Satti I Meyer J Harris SA Manjaly Thomas ZR Griffiths K Antrobus RD. Safety and immunogenicity of a candidate tuberculsis vaccine MVA85A delivered by aerosol in BCG-vaccinated healthy adults: a phase 1, double-blind, randomised controlled trial. Lancet Infect Dis. (2014) 14:939–46. 10.1016/S1473-3099(14)70845-X 25151225 PMC4178237

[53] Bloom B R. New Promise for Vaccines against Tuberculosis. N Engl J Med. (2018) 379:1672–4. 10.1056/NEJMe1812483 30252629

[54] Vekemans J Gebreselassie N Zignol M Friede M Kasaeva T Swaminathan S A new tuberculsis vaccine: breakthrough, challenges, and a call for collaboration. Lancet Infect Dis. (2019) 19:123–5. 10.1016/S1473-3099(19)30003-9 30712831

