# Supplementary material for: Immunogenicity and Safety of the M72/AS01E Candidate Vaccine Against Tuberculosis: A Meta-Analysis
Source: Front Immunol. 2019 Sep 3;10:2089. doi: 10.3389/fimmu.2019.02089 (PMC6735267; doi:10.3389/fimmu.2019.02089)
Supplement: Supplementary file 1 [file Data_Sheet_1.docx]

**Supplemental Material 1. Text and MeSH search terms**

**PubMed**
((((((tuberculosis vaccine[MeSH Major Topic])) OR (M72/AS01E[Title/ Abstract])) OR (M72/AS01[Title/Abstract]))) AND ((immuno* or safe*))) AND (English[Language])

**Embase**

('tuberculosis vaccine'/exp/mj OR 'm72/as01e': ti,ab,kw OR'm72/as01':ti,ab, kw) AND (immuno* OR safe*) AND english:la

**Cochrane Library**

(tuberculosis vaccine or M72/AS01E or M72/AS01): ti,ab, kw AND (immuno* OR safe*)
