# Supplementary material for: Immunogenicity and Safety of the M72/AS01E Candidate Vaccine Against Tuberculosis: A Meta-Analysis
Source: Front Immunol. 2019 Sep 3;10:2089. doi: 10.3389/fimmu.2019.02089 (PMC6735267; doi:10.3389/fimmu.2019.02089)
Supplement: Supplementary file 3 [file Data_Sheet_3.docx]

**Supplemental material 3. Methodological quality & risk of bias**

**
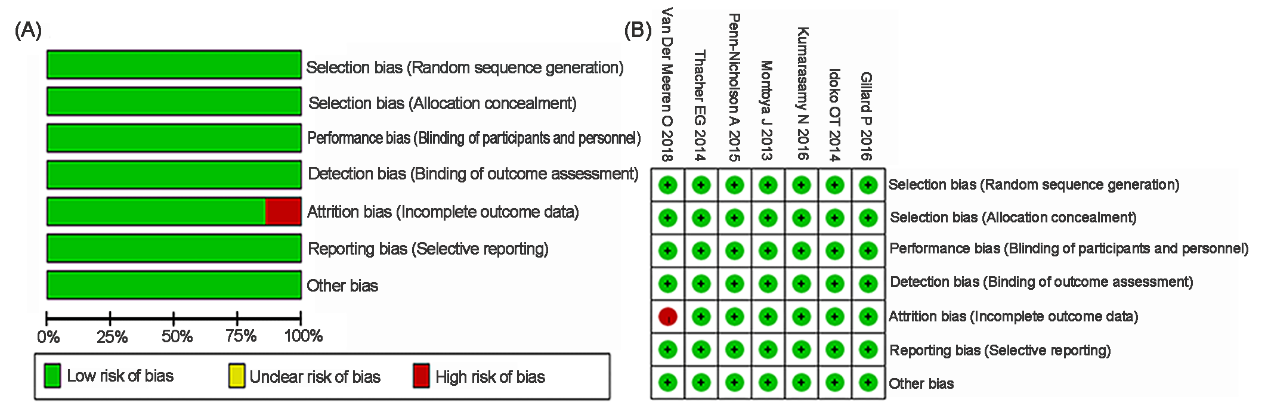
**

**Methodological quality & risk of bias.** (A)Risk of bias graph, a plot of the distribution of investigators’ judgements across studies for each risk of bias item. The figure illustrates the proportion of studies with each of the judgements (i.e., ‘low risk’, ‘high risk’, ‘unclear risk’) for each entry in the tool. (B)Risk of bias summary, a summary table of investigators’ judgements for each risk of bias item in each study. The figure presents all judgements in a cross-tabulation of study.
